# Epithelioid inflammatory myofibroblastic sarcoma: a clinicopathological, immunohistochemical and molecular cytogenetic analysis of five additional cases and review of the literature

**DOI:** 10.1186/s13000-016-0517-z

**Published:** 2016-07-27

**Authors:** Lin Yu, Jinguo Liu, I Weng Lao, Zhiguo Luo, Jian Wang

**Affiliations:** 1 Department of Pathology, Fudan University Shanghai Cancer Center, Fudan University, 270 Dong An Road, Shanghai, 200032 China; 2 Department of Medical Oncology, Fudan University Shanghai Cancer Center, Fudan University, Shanghai, 200032 China; 3 Department of Oncology, Shanghai Medical College, Fudan University, Shanghai, 200032 China; 4 Department of Pulmonary Medicine, Zhongshan Hospital, Fudan University and Shanghai Respiratory Research Institute, Shanghai, 200032 China

**Keywords:** Gastrointestinal tract, Soft tissue tumor, Inflammatory myofibroblastic tumor, RANBP2-ALK, Fluorescence in situ hybridization

## Abstract

**Background:**

To explore the clinical characteristics and pathological features of epithelioid inflammatory myofibroblastic sarcoma (EIMS) with emphasis on the diagnostic spectrum.

**Methods:**

The clinical data and histological features in 5 additional cases of EIMS were retrospectively reviewed. Immunohistochemical study and interphase fluorescence in situ hybridization (FISH) analysis were carried out.

**Results:**

There were 2 males and 3 females with age at presentation ranging from 15 to 58 years (mean, 37 years). All 5 tumors were intra-abdominal with 2 arising in the mesentery and 1 each in the omentum, rectum and transverse colon. The tumor size ranged from 5 to 20 cm in maximum diameter (mean, 10.7 cm). Histologically, all 5 tumors were composed predominantly of large epithelioid cells possessing vesicular nuclei, prominent nucleoli, and amphophilic cytoplasm. Mitotic figures were easily identified (mean, 20/10HPF). Tumor cells were arranged in clusters or sheets embedded in a myxoid stroma containing prominent neutrophils. A minor component of spindle cells was present in focal areas. By immunohistochemistry, all 5 cases were positive for anaplastic lymphoma kinase (ALK) with a nuclear membrane pattern in 4 and cytoplasmic staining with perinuclear accentuation in 1. Besides ALK, tumor cells stained variably for desmin (4/5), alpha smooth muscle actin (2/5), muscle-specific actin (1/2) and pan-cytokeratin (1/4). FISH analysis demonstrated the presence of ALK rearrangement in all 5 cases. Of 5 patients, 3 developed local recurrence, 1 died of disease 8 months after surgery.

**Conclusion:**

EIMS represents a highly aggressive variant of inflammatory myofibroblastic tumor characterized by epithelioid morphology, prominent neutrophilic infiltrate, and nuclear membrane staining of ALK with ALK rearrangement. As patients with ALK-rearrangement tumors may benefit from targeted therapy, accurate diagnosis of EIMS is very important. Familiar with the characteristic features of EIMS will help pathologists avoid misdiagnosing the tumor as other malignancies.

## Background

Inflammatory myofibroblastic tumor (IMT) is a distinct mesenchymal neoplasm composed of spindled fibroblastic and myofibroblastic cells in a myxoid to collagenous stroma containing abundant lymphocytic and plasmacytic inflammatory infiltrate [1]. IMT usually affects children and adolescents, although a broad age range has been documented. The most common anatomical locations are the abdominopelvic region, lung, mediastinum and retroperitoneum [1]. Approximately 50 % of IMTs aberrantly express *anaplastic lymphoma kinase* (ALK) protein triggered by clonal rearrangements of ALK gene located on chromosome 2p23 [2]. IMT is considered to be a soft tissue tumor with an intermediate biological behavior. However, a small percentage of cases behave aggressively [3].

In 2011, Mariño-Enríquez et al. [4] described a novel variant of IMT, which they nominated as epithelioid inflammatory myofibroblastic sarcoma (EIMS). In contrast to the classic IMT, EIMS is characterized by an epithelioid morphology accompanied with prominent neutrophilic inflammatory infiltrate. Clinically, it also differs from the classic IMT by a more aggressive behavior with short disease-free survival. Recognizing EIMS as a distinct variant of IMT is very important as patients with ALK-rearrangement EIMS may benefit from targeted therapy. As EIMS has not been widely recognized, we present here 5 additional cases of EIMS with a clinicopathological, immunohistochemical and molecular cytogenetic analysis.

## Methods

Five cases of EIMS were retrieved from the archive files of the Department of Pathology, Fudan University Shanghai Cancer Center. The cases were initially diagnosed between 2012 and 2015 and were all consultation cases. The clinical and follow-up data were obtained from the electronic medical records, hospital discharge summary or by telephone inquiry. All available hematoxylin-and-eosin (H&E) slides were reassessed for cytomorphology, mitotic activity, composition of inflammatory infiltrate, stromal change, and presence of necrosis.

Immunohistochemical study was performed on paraffin-embedded sections on Ventana Benchmark XT autostainer (Roche). The primary antibodies used in the study included desmin (D33, dilution 1:500; DAKO), alpha smooth muscle actin (1A4, dilution 1:400; DAKO), muscle-specific actin (HHF-35, dilution 1:400; DAKO), H-caldesmon (h-CALD, dilution 1:400; DAKO), ALK (5A4, dilution 1:100; DAKO), CD30 (Ber-H2, dilution 1:50; DAKO), vimentin (V-9, dilution 1:200; DAKO), S100 protein (polyclonal, dilution 1:300; DAKO), pan-cytokeratin(AE1/AE3, dilution 1:100; DAKO), epithelial membrane antigen (E29,dilution 1:150; DAKO), myogenin(MYF4, dilution 1:500; Novocastra), CD117 (polyclonal, dilution 1:100; DAKO), discovered on GIST-1 (DOG1) (SP31, dilution 1:100; DAKO), CD34(QBEnd/10, dilution 1:50;DAKO) and Ki-67(MIB-1, dilution 1:150; DAKO). Appropriate positive and negative controls were run simultaneously for all antibodies tested.

Interphase fluorescence in situ hybridization analysis was carried out on 5-μm-thick sections of formalin-fixed, paraffin-embedded tissue in 5 cases, according to the manufacturer’s protocol. The presence of ALK gene rearrangement at 2p23 was tested using the LSI ALK dual-color break-apart probe (Abbott Molecular, Vysis, Des Plaines, IL). The fluorescence signals were analyzed using an Olympus BX51 fluorescence microscope (Olympus, Tokyo, Japan). A total of 100 nuclei were evaluated from each specimen.

## Results

### Clinical history

#### Case 1

**Fig. 1:**
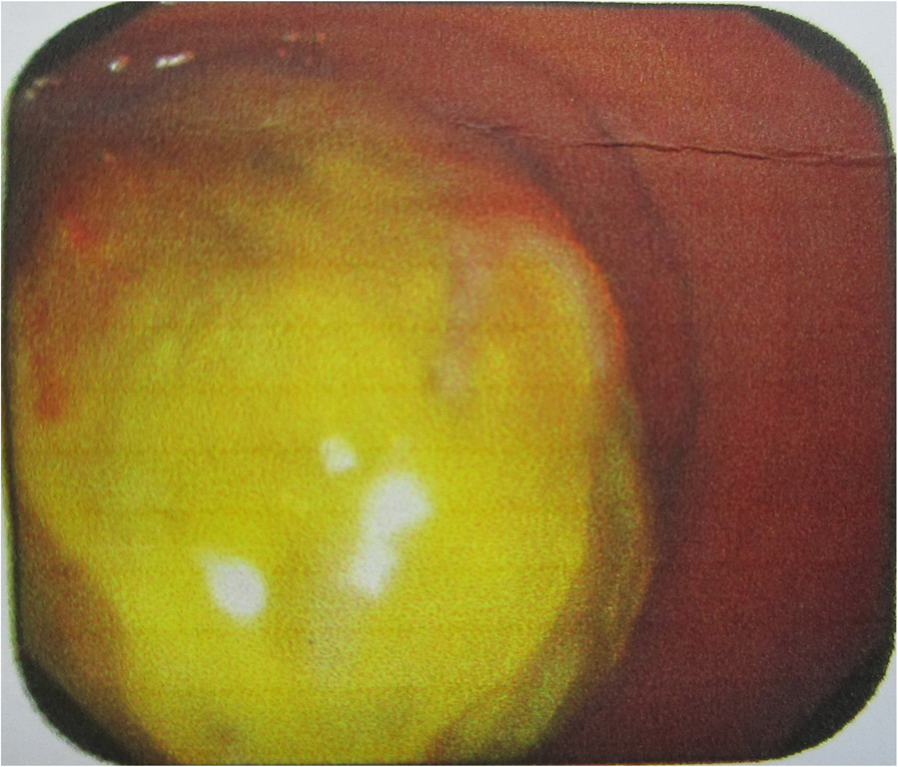
Endoscopic appearance of EIMS. Colonoscopy examination showed a polypoid mass protruding into the rectum cavity

A 37-year-old woman went to the clinic of a local hospital because of abdominal pain and hematochezia for one week. Colonoscopy examination showed a polypoid mass protruding into the rectum cavity, measuring approximately 5 cm in maximum diameter, with a rough and uneven surface (Fig. 1). Then the patient underwent a partial rectectomy. The lesion was considered as a myogenic sarcoma, preferring pleomorphic leimyosarcoma by the referring pathologists. The postoperativ adjunctive therapy was not administrated . There was no evidence of recurrence or metastasis 8 months after surgery.

#### Case 2

**Fig. 2:**
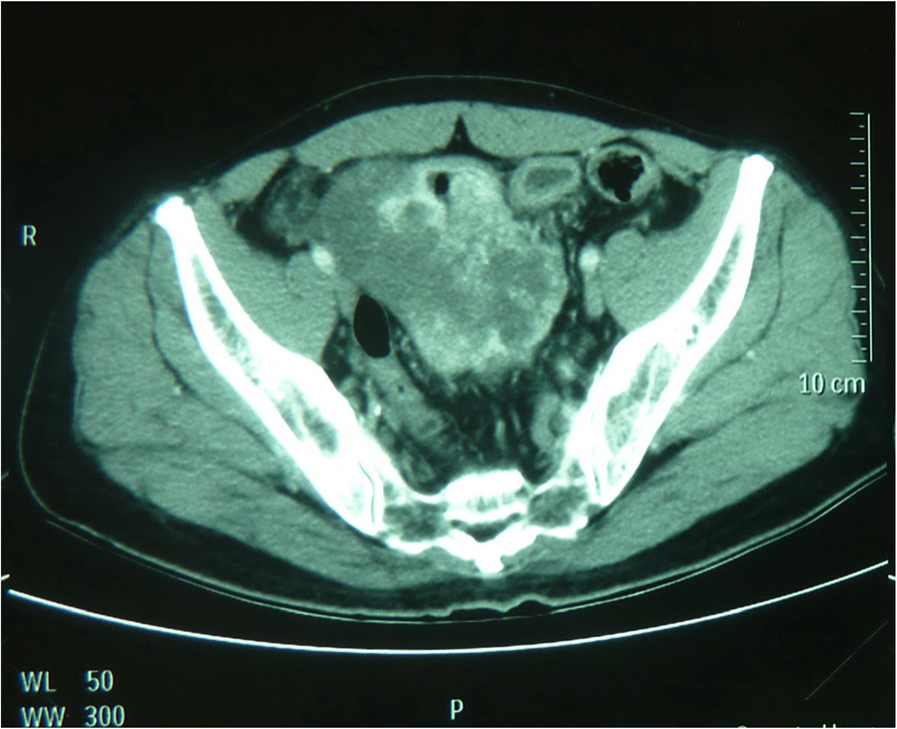
Imaging features of EIMS. Computed tomography (CT) scan revealed an enormous heterogeneous mass in the pelvic cavity

The patient was a 55-year-old man who complained of abdominal pain and distension lasting for two days. Physical examination showed abdominal mass with tenderness. No obvious mass was found by colonoscopy. X-ray displayed intestinal obstruction. Computed tomography (CT) scan demonstrated a solid mass in the pelvic cavity (Fig. 2). Clinically, the lesion was suspicious of gastrointestinal stromal tumor (GIST). Intraoperatively, the mass was found on the terminal ileum nearing the ileocecal junction. Partial ileal resection and appendectomy with intestinal anastomosis were performed shortly after. The lesion was originally diagnosed as malignant mesothelioma by the referring pathologist. After reevaluation of the H&E slides with application of immunohistochemistry and FISH analysis, the diagnosis of EIMS was finally rendered. The patient developed local recurrence 2 months after surgery. A re-excision of the recurrent tumor with adjuvant chemotherapy was performed. He was free of disease at 10-month follow up.

#### Case 3

A 22-year-old young man presented with abdominal mass with intermittent abdominal pain for eight days and fever for five days. Subsequent CT scan revealed a solid heterogeneous mass of the right abdominal cavity, suspicious of GIST. After the admission, the patient underwent a complete excision of the abdomen mass and partial transverse colectomy soon. Intraoperatively, an enormous mass measuring approximately 20 × 15 cm was identified, with multiple small omental or mesenteric nodules. Pathological examination of the submitted specimen showed that malignant tumor was suspected as GIST. To confirm the diagnosis, the pathological materials were sent to our department for further consideration. With the adjunctive study of immunohistochemistry and FISH, the lesion was re-diagnosed as EIMS. The patient developed local recurrence 2 months after surgery. The targeted therapy of ALK inhibitor (crizotinib) was administrated later. He achieved a partial response to the treatment. He was alive with disease at 14-month follow up.

#### Case 4

The patient was a 58-year-old woman who complained of a slowly enlarging and painless mass on the abdomen for two months. Physical examination showed an ill-defined solid mass of the right lower abdominal wall with no tenderness. Then she received a local resection. Intraoperative examination revealed that the mass was firm in consistency and infiltrated into the skeletal muscle of the abdomen wall. Pathologically, the tumor was considered as pleomorphic sarcoma by the referring pathologist. After surgery, the patient was treated with the additional chemotherapy. She developed local recurrence 2 months later and died of disease at 8-month follow-up.

#### Case 5

A 15-year-old young woman complained of recurrent abdominal pain for four months and abdominal mass for four days. Physical examination revealed a well-defined solid mass of the right abdomen with mild tenderness. Ultrasonography displayed an abdominal tumor with heterogeneous internal echoes, measuring about 10.2 × 7.5 cm. Subsequently, the patient received a complete excision of the abdomen mass and partial transverse colectomy. During the operation, the tumor was identified on the transverse colon and measured approximately 12 × 8 cm, with multiple enlarged omental or mesenteric lymph nodes. The lesion was considered as fibrosarcoma with inflammatory cell infiltrates by the referring pathologist. After surgery, the patient did not receive the adjunctive therapy. She was free of disease at 7-month follow up.

### Pathologic features

Tumor size ranged from 5 to 20 cm in maximum diameter (mean, 10.7 cm). On cut section, the tumors were grayish-white to pink-tan in color, moderate or fleshy in consistency, with myxoid appearance in some areas.

**Fig. 3:**
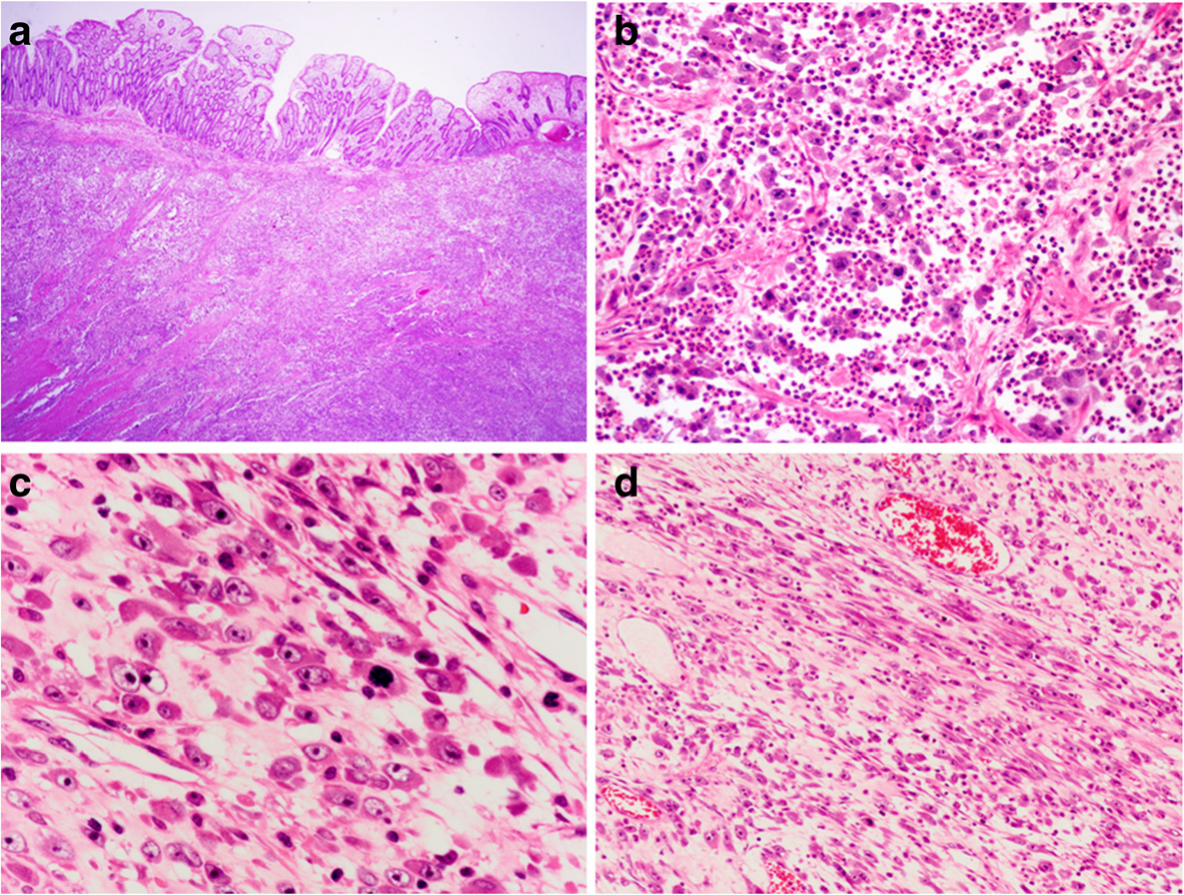
Histological features of EIMS. **a** A mesenteric tumor infiltrated the adjacent small bowel wall (40 × original magnification). **b** The tumor was composed predominantly of sheets of round-to-epithelioid cells with a prominent inflammatory infiltrate (200 × original magnification).**c** The epithelioid tumor cells showed vesicular nuclei, large and prominent nucleoli, and amphophilic-to-basophilic cytoplasm (400 × original magnification).**d** A minor spindle cell component was present in all cases (100 × original magnification)

On low power, the tumor displayed a polypoid appearance in case 1, which was mainly located on the submucosa and infiltrating the muscularis. In case 2 and 3, the masses invaded the intestinal wall (Fig. 3a). In case 5, the tumor was located on the submucosa and muscularis and invaded into the serosa. Tumor cells were predominantly composed of non-cohesive sheets or clusters of round to epithelioid cells embedded in a loose or myxoid stroma containing abundant neutrophilic inflammatory infiltrates (Fig. 3b). On high power, tumor cells possessed large vesicular nuclei with prominent nucleoli and amphophilic cytoplasm (Fig. 3c). The mitotic figures were easily encountered with a mean count of 20/10 HPF (range, 12-35/10HPF). Atypical forms were also present. There was a minor component of spindle cells comprising less than 10 % of the tumor in all cases (Fig. 3d). The stroma was predominantly myxoid in 4 cases, and collagenous in 1. Focal necrosis was present in 3 cases. Lymphovascular invasion was seen in case 5.

### Immunohistochemical and molecular findings

**Fig. 4:**
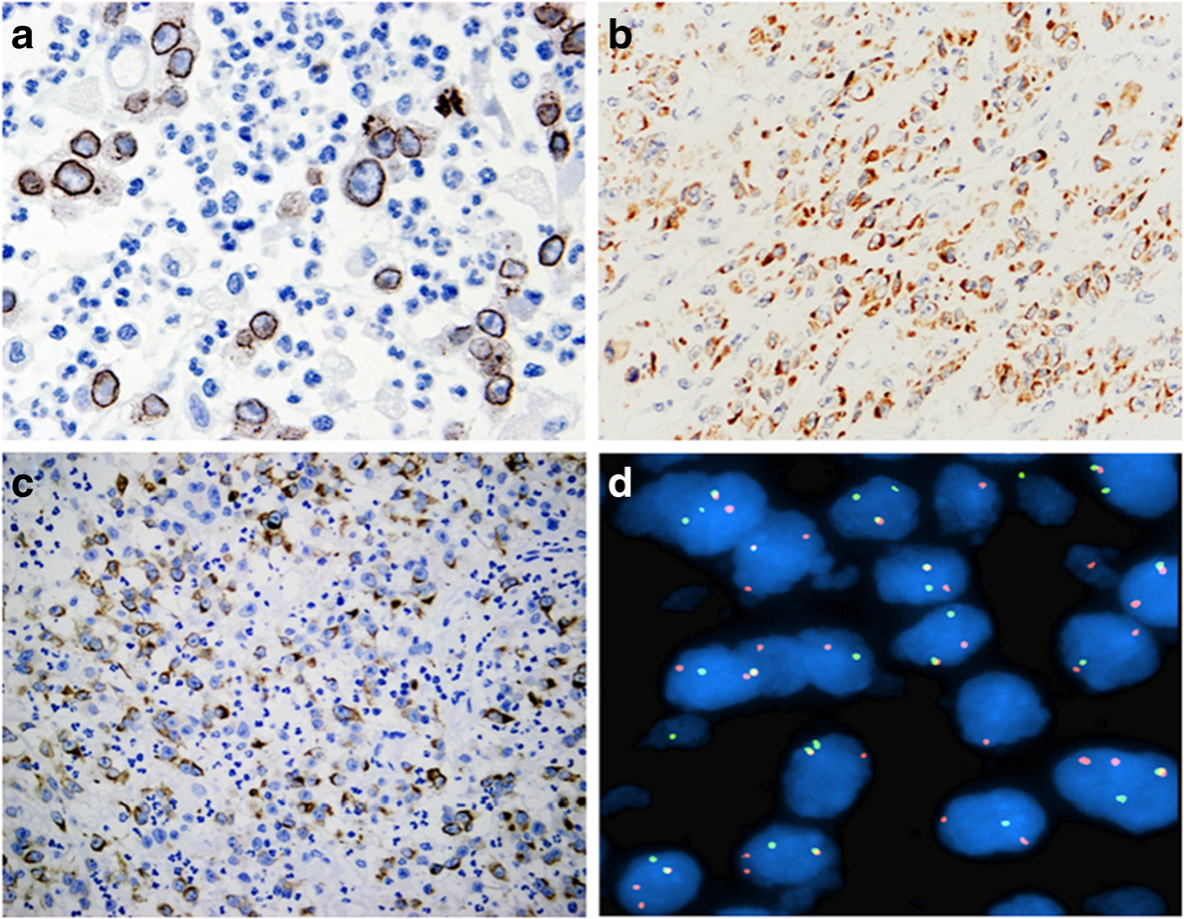
Immunohistochemical and genetic features of EIMS. **a** Immunohistochemistry for ALK showed a nuclear membrane staining pattern (400 × original magnification). **b** Case 3 showed ALK cytoplasmic staining with perinuclear accentuation (200 × original magnification). **c** Diffuse and strong positivity for desmin (200 × original magnification). **d** Fluorescence in situ hybridization (FISH) showed split apart of the red and green signals, confirming the presence of ALK rearrangement (1000 × original magnification)

Immunohistochemically, all 5 tumors were strongly positive for ALK, with nuclear membrane pattern in 4 cases (Fig. 4a), and cytoplasmic staining with perinuclear accentuation fashion in 1case (Fig. 4b). Besides ALK, tumor cells also showed variable expression of desmin (4/5) (Fig. 4c), smooth muscle actin (2/5), muscle-specific actin (1/2) and AE1/AE3 (1/5). Other markers, including CD30, h-caldesmon, myogenin, epithelial membrane antigen, CD117, DOG1, CD34 and S-100 protein, were all negative. Ki-67 index was 15 % ~ 50 % (mean, 30 %).

By FISH analysis, all 5 tumors showed ALK gene rearrangement, as indicated by the presence of isolated green (centromeric) and orange (telomeric) signals flanking the ALK locus at 2p23 (Fig. 4d).

## Discussion

IMT is a well-described entity with a predilection for children and adolescents and occurs predominantly in the upper respiratory tract, lung and abdomen. Histologically, it is composed of spindled fibroblasts and myofibroblasts with occasional “ganglion-like” cells present in the tumor. IMT with prominent epithelioid cytomorphology is very rare. In a study with 73 cases of IMT, Cook et al. found 4 cases that exhibited round cell transformation characterized by large polygonal cells with large nuclei and prominent nucleoli, in a loose, pale staining background [5]. In contrast to classic IMT, these four cases displayed more aggressive behavior. They proposed for the first time the nomenclature of “round cell transformation” to emphasize this distinctive feature. One of these 4 cases showed distinctive nuclear membrane staining pattern of ALK. Subsequent studies further demonstrated that this distinctive nuclear membrane staining pattern of ALK corresponded to ALK-RANBP2 fusions [6-8]. In 2010, Butrynski et al. reported sustained partial response to the ALK inhibitor crizotinib in a patient with ALK-translocated IMT with epithelioid cytomorphology [9]. Their report indicated that this aggressive form of IMT merited a separation from the classic IMT. In 2011, Marino-Enriquez et al. [4] described the clinicopathologic, immunohistochemical and genetic features of 11 additional cases in detail and proposed the designation as EIMS to highlight both the distinct morphology and malignant behavior of this aggressive form of IMT.

As EIMS has not been widely recognized, the incidence remains underestimated. To the best of our knowledge, only about 20 cases have been documented in the English literature [4–12]. In this study, we describe a small series of 5 additional cases, increasing the number of reported EIMS to 25. The clinical features of these EIMSs are summarized in Table 1. Of these 25 cases, 18(72 %) patients were adults, and the other 7 patients were children and adolescents. Overall, the median and average ages of patients with EIMS were 34 and 30.7 years, respectively (range, 7 months-63 years). There was a prominent male predilection with a male to female ratio of 5.3:1. With regard to the location distribution, 21(84 %) tumors occurred in the intra-abdominal sites, including the mesentery, omentum, peritoneum, abdominal cavity and retroperitoneum, 3 in the organs (1 each in liver, rectum and transverse colon), and another 1 in the pleural cavity. The tumor size (unavailable in 5 cases) ranged from 5 to 26 cm in maximum diameter, with an average size of 13.2 cm.10 tumors were multifocal at the time of diagnosis, consisting of a large dominant mass and multiple small omental, mesenteric or peritoneal nodules. Clinically, patients usually manifested with abdominal pain or masses, sometimes with ascites.

**Table 1:** Clinical features of 25 cases of epithelioid inflammatory myofibroblastic sarcoma

Case	Age/Sex	Site	Size (cm)	Multifocal	Treatment	Recurrence	Metastasis	Follow up	Source
1	7y/M	Abdominal cavity	NA	No	SE, CT	Yes (5w, 5 m)	No	NED(5 m)	Ma et al. [7]
2	7 m/M	Mesentery and omentum	11	Yes	SE	Yes (8 m)	No	AWD(8 m)	
3	2y/M	Retroperitoneal and abdominal cavity	10	No	SE	No	No	NED(36 m)	Patel et al. [6]
4	34y/M	Liver	8	No	SE	Yes (5 m)	No	DOD(5.5 m)	Chen et al. [8]
5	44y/M	Omentum	NA	Yes	SE, CT, ALKi	Yes (5 m)	Liver (12 m)	AWD(39 m)	Butrynski et al. [9]
6	41y/M	Omentum	26	Yes	ST, CT, ALKi	Yes (12 m)	Liver(12 m)	NED(40 m)	Marino-Enriquez et al. [4]
7	59y/M	Mesentery of small bowel	15	Yes	SE, CT	Yes	No	DOD (12 m)	
8	6y/M	Omentum	10.5	No	SE, CT	Yes	No	AWD(3 m)	
9	28y/M	Mesentery of small bowel	NA	Yes	NA	NA	NA	NA	
10	63y/M	Mesentery of small bowel	25	No	SE, CT	Yes	No	DOD(3 m)	
11	42y/M	Intra-abdominal	NA	No	SE, CT	Yes	No	AWD(13 m)	
12	7 m/M	Peritoneum	10	No	SE, CT, RT	Yes	No	DOD(36 m)	
13	40y/M	Peritoneum	8	No	SE, CT, RT	Yes	Lung, liver, and lymph node		
14	31y/M	Mesentery of small bowel	17.5	Yes	SE, CT	Yes	No	DOD(11 m)	
15	6y/M	Omentum and mesentery	14	Yes	SE	NA	NA	NA	
16	39y/M	Mesentery of small bowel	15	Yes	SE	NA	NA	NA	
17	57y/M	Pleura or chest wall	NA	NA	ALKi	NA	NA	NA	Kozu et al. [10]
18	19y/F	Mesentery of small bowel	19	No	SE	Yes(9w)	No	DOD(12w)	Li et al. [11]
19	39y/M	Mesentery of colon	15	No	SE, CT	Yes(4 m)	No	AWD(12 m)	
20	22y/M	Mesentery of small bowel	6	Yes	SE, CT, ALKi	Yes(3 m, 4 m)	No	AWD(14 m)	Kimbara et al. [12]
21	37/F	Rectum	5	No	SE	No(8 m)	No	NED(8 m)	Current cases
22	55/M	Mesentery of ileum	11	No	SE, CT	Yes(2 m)	No	NED(10 m)	
23	22/M	Mesentery of colon	20	Yes	SE, ALKi	Yes(2 m)	No	AWD(14 m)	
24	58/F	Omentum	5.5	No	SE, CT	Yes(2 m)	No	DOD(8 m)	
25	15/F	Transverse colon	12	No	SE	No(7 m)	No	NED(7 m)	

*ALKi* ALK inhibitor, *AWD* alive with disease, *CE* cholecystectomy, *CT* chemotherapy, *DOD* dead of disease, *NA* not available, *NED* no evidence of disease, *RT* radiation therapy, *SE* surgical excision

EIMS has distinctive morphological features. The tumor is typically characterized by loosely arrayed, round or epithelioid neoplastic cells with vesicular nuclei, prominent large nucleoli and amphophilic to eosinophilic cytoplasm distributed in a widespread myxoid stroma. The striking feature of EIMS is the presence of obvious inflammatory infiltrates frequently composed of neutrophils. All tumors almost contained a small amount of spindle cell component. Immunohistochemically, EIMS demonstrated a unique nuclear membrane staining pattern of ALK, which was observed in 80 % (20/25) of cases (Table 2). However, several cases (20 %, 5/25) showed ALK perinuclear or cytoplasmic staining pattern. Another diagnostic immunophenotype is diffuse and strong expression of desmin in almost all cases (86.4 %, 19/22). In addition, the tumor displayed variable expression of CD30 (61.1 %, 11/18), alpha smooth muscle actin (47.4 %, 9/19) and cytokeratin (15.8 %, 3/19).

**Table 2:** Immunohistochemical and genetic features of 25 cases of epithelioid inflammatory myofibroblastic sarcoma

Case	Immunohistochemical features								ALK		
	desmin	SMA	H-caldesmon	CD30	CK	EMA	S-100	myogenin	IHC	FISH	RT-PCR
1	NA	NA	NA	NA	NA	NA	NA	NA	NM+	+	RANBP2-ALK
2	NA	NA	NA	NA	NA	NA	NA	NA	NM+	+	RANBP2-ALK
3	+	+	-	NA	+	NA	-	NA	PN+	+	RANBP2-ALK
4	-	-	NA	-	-	NA	-	NA	NM+	NA	RANBP2-ALK
5	+	-	NA	+	-	-	-	-	NM+	+	RANBP2-ALK
6	+	-	-	+	-	-	-	-	NM+	+	RANBP2-ALK
7	+	+	NA	NA	-	NA	-	-	NM+	+	NA
8	+	-	NA	NA	-	NA	-	-	NM+	+	NA
9	+	+	NA	+	-	-	-	NA	NM+	NA	NA
10	+	-	-	+	-	-	-	-	PN+	+	NA
11	+	NA	NA	NA	NA	NA	NA	NA	NM+	NA	NA
12	+	NA	-	+	NA	NA	NA	-	PN+	+	NA
13	-	+	-	+	-	-	-	-	NM+	+	NA
14	+	-	-	+	-	NA	-	-	NM+	+	NA
15	+	NA	-	+	NA	NA	NA	NA	NM+	+	RANBP2-ALK
16	+	+	-	+	-	NA	-	-	NM+	+	RANBP2-ALK
17	+	-	NA	-	+	NA	-	NA	PN+	+	NA
18	+	+	-	+	-	-	-	-	NM+	+	NA
19	+	+	-	+	-	-	-	-	NM+	+	RANBP2-ALK
20	NA	NA	NA	NA	NA	NA	NA	NA	NM+	NA	RANBP2-ALK
21	+	-	-	-	-	NA	-	-	NM+	+	NA
22	+	-	NA	-	-	-	-	-	NM+	+	NA
23	+	-	-	-	-	-	-	-	PN+	+	NA
24	+	Focal +	-	-	Focal +	NA	-	-	NM+	+	NA
25	-	+	NA	-	-	-	-	-	NM+	+	NA
Total	86 % (19/22)	47 % (9/19)	0 % (0/13)	61 % (11/18)	16 % (3/19)	0 % (0/10)	0 % (0/19)	0 % (0/16)	100 % (25/25)	100 % (21/21)	100 % (10/10)

*CK* cytokeratin, *EMA* epithelial membrane antigen, *FISH* fluroscence in situ hybridization, *IHC* immunohistochemistry, *NA* not avaiable, *NM* nuclear membrane staining, *PN* cytoplasmic staining with perinuclear accentuation, *RT-PCR* reverse transcription polymerase chain reaction, *SMA* smooth muscle actin

Approximately 50 % of IMTs aberrantly express ALK protein triggered by clonal rearrangements of ALK gene located on chromosome 2p23 [3]. A variety of ALK partner genes have been subsequently identified in IMT owing to diverse chromosomal rearrangements, including TPM3 at 1p23, TPM4 at 19p13, CLTC at 17q23, CARS at 11p15, ATIC at 2q35, SEC31L1 at 4q21, PPFIBP1 at 12p11 and ran-binding protein 2 (RANBP2) at 2q13 [13–17]. Different fusion partners may result in distinct ALK staining pattern when detected by ALK antibody. IMT with TPM3, TPM4, CARS, ATIC, and SEC31L1 fusion often shows diffuse cytoplasmic staining of ALK [5, 6], whereas with CLTC fusion, displays granular cytoplasmic staining [14]. By comparison, EIMS harbors a specific RANBP2-ALK fusion genes resulting from t (2;2) (2q12;2p23). All 21 cases tested by FISH so far showed positive signal of ALK translocation. Of note, all 10 cases in which the cDNA fusions were examined by reverse transcription-polymerase chain reaction (RT-PCR) constantly presented identical fusion points between exon 18 of RANPB2 and exon 20 of ALK (Table 2). The fusion partner of RANBP2 encodes a nuclear pore protein, attributing to the nuclear membrane or perinuclear staining pattern in EIMS. 9 cases except for one confirmed by RT-PCR in previous studies reported to contain RANBP2-ALK fusion showed this distinct staining pattern [4-9, 11, 12]. The biology function of the RANBP2-ALK fusion protein remains largely unknown. Several studies discovered that the chimeric RANPB2-ALK gene could promote cell growth and proliferation independent of cytokine in vitro [18, 19]. Furthermore, nearly all reported cases containing RANBP2-ALK fusion gene pursued an aggressive behavior [4, 5, 7, 9, 11, 12]. Accordingly, we infer that RANPB2-ALK fusion gene might be a potential molecular mechanism for the rapid growth and recurrence of EIMS. However, further investigations are still needed to establish the relationship this fusion gene with aggressive clinical course of EIMS.

EIMS can be diagnosed in combination with morphologic features and an appropriate immunohistochemical panel. Nonetheless, it is possibly risky to diagnose EIMS rely solely on the histology or ALK expression, because not all IMTs with epithelioid/round cell morphology carry the genetic alteration of EIMS [5], as well as ALK cytoplasmic staining could be encountered in a few mesenchymal tumors, such as rhabdomyosarcoma, malignant peripheral nerve sheath tumor, leiomyosarcoma, lipogenic tumors, and Ewing sarcoma/ peripheral primitive neuroectodermal tumor [20, 21]. Therefore, for those cases with atypical morphology or immunoprofile, we recommend further genetic detection of ALK rearrangement by FISH or RT-PCR to confirm the diagnosis of EIMS.

A variety of tumors should be included into the differential diagnoses of EIMS. Anaplastic large cell lymphoma (ALCL) is the most likely to be confused with EIMS, because both tumors have an overlapping immunophenotype and cytogenetic feature, including reactivity for CD30, ALK and SMA, and the presence of ALK rearrangement [22]. Detection of ALK translocation by FISH would not be helpful in the distinction of both tumors. However, strong expression of desmin and nuclear membrane ALK staining are not observed in ALCL. Moreover, ALCL is absent of RANBP2-ALK fusion gene in EIMS, which can be detected by RT-PCR. In addition, epithelioid leiomyosarcoma also needs to be differentiated from EIMS, but the former usually contains at least focal areas of typical spindle cell leimyosarcoma and is lacking of ALK nuclear membrane expression. Other tumors with round or epithelioid morphology may also be discriminated from EIMS, such as solid variant of alveolar rhabdomyosarcoma, epithelioid gastrointestinal stromal tumor, epithelioid malignant peripheral nerve sheath tumor, myxoid/round cell liposarcoma, myxofibrosarcoma, poorly differentiated carcinoma, and melanoma. However, these entities can be easily distinguished by their lack of nuclear membrane ALK staining and their respective morphology and immunophenotypes.

With respect to the biologic behavior of EIMS, 8 of 21 patients with follow-up information died of the disease (6 within 1 year of diagnosis, 2 within 3 years of diagnosis), 7 was alive with disease, and the other 6 remained well with no evidence of disease [4-9, 11, 12]. The median time of overall survival was 11 months (mean, 15.4 months). Furthermore, 18 patients experienced local recurrence, and 3 developed distant metastases (2 to liver, 1 to lung, liver and lymph node) [4, 5, 7, 9, 11, 12]; its local recurrence rate (86 %, 18/21) and distant metastasis rate (14 %, 3/21) are both markedly higher than those of the conventional IMT. The data indicate that EIMS pursues a more aggressive biologic behavior than the usual IMT. Up to present, the clinicopathologic factors related to prognosis of EIMS is still unknown. Several studies suggested that its abdominopelvic origination, bigger tumor size, epithelioid morphology and RANBP2-ALK are possibly related to its aggressive course [4, 5, 7, 9, 11, 12, 23].

The optimal therapy of EIMS has not been well established. A surgical resection remains to be considered as the mainstay of treatment. In view of postoperative adjuvant therapy, available experiences are very limited. Most of reported cases are administrated by postoperative chemotherapy or radiotherapy, but it seems to have no obvious effects in the control of rapid recurrence [4, 7, 9, 11, 12]. Recently, ALK inhibitor, crizotinib, has been applied in the treatment of EIMS with certain effectiveness [4, 9, 10, 12, 24]. Thus, crizotinib might serve as a provisional adjuvant in the therapy of EIMS. In our own series, case 3 was treated with ALK inhibitor (crizotinib) after the recurrence. Follow-up CT scan showed that the residual tumor partially shrinked in size. The patient felt well after four courses of treatment.

## Conclusion

In summary, EIMS is a highly aggressive IMT variant characterized by epithelioid cytomorphology, often neutrophilic inflammatory infiltrate, loose or myxoid stroma and distinctive nuclear membrane or perinuclear ALK staining. It has a striking male predominance and mostly arises in the intra-abdominal locations. Familiarity with histologic features of this special tumor type with application of an appropriate immunohistochemical panel can arrive to a correct diagnosis. Detection of ALK rearrangement by FISH or RT-PCR will further confirm the diagnosis of EIMS and provide a reliable reference for ALK-targeted therapy.

## Abbreviations

ALCL, anaplastic large cell lymphoma; ALK, anaplastic lymphoma kinase; CT, computed tomography; EIMS, epithelioid inflammatory myofibroblastic sarcoma; FISH, fluorescence in situ hybridization; GIST, gastrointestinal stromal tumor; H&E, hematoxylin-and-eosin; IMT, inflammatory myofibroblastic tumor (IMT); RT-PCR, reverse transcription-polymerase chain reaction
